# An investigation into aripiprazole’s partial D_2_ agonist effects within the dorsolateral prefrontal cortex during working memory in healthy volunteers

**DOI:** 10.1007/s00213-016-4234-9

**Published:** 2016-02-22

**Authors:** Anna Murphy, Serdar Dursun, Shane McKie, Rebecca Elliott, John Francis William Deakin

**Affiliations:** Neuroscience and Psychiatry Unit, Institute of Brain, Behaviour and Mental Health, University of Manchester, G.708 Stopford Building, Manchester, M13 9PT UK; Department of Psychiatry, University of Alberta, Edmonton, Alberta Canada

**Keywords:** Working memory, Aripiprazole, Risperidone, n-Back, Dopamine, Dorsolateral prefrontal, Antipsychotic, Partial agonism, D2

## Abstract

**Rationale:**

Working memory impairments in schizophrenia have been attributed to dysfunction of the dorsolateral prefrontal cortex (DLPFC) which in turn may be due to low DLPFC dopamine innervation. Conventional antipsychotic drugs block DLPFC D_2_ receptors, and this may lead to further dysfunction and working memory impairments. Aripiprazole is a D_2_ receptor partial agonist hypothesised to enhance PFC dopamine functioning, possibly improving working memory.

**Objectives:**

We probed the implications of the partial D_2_ receptor agonist actions of aripiprazole within the DLPFC during working memory. Investigations were carried out in healthy volunteers to eliminate confounds of illness or medication status. Aripiprazole’s prefrontal actions were compared with the D_2_/5-HT_2A_ blocker risperidone to separate aripiprazole’s unique prefrontal D_2_ agonist actions from its serotinergic and striatal D_2_ actions that it shares with risperidone.

**Method:**

A double-blind, placebo-controlled, parallel design was implemented. Participants received a single dose of either 5 mg aripiprazole, 1 mg risperidone or placebo before performing the n-back task whilst undergoing fMRI scanning.

**Results:**

Compared with placebo, the aripiprazole group demonstrated enhanced DLPFC activation associated with a trend for improved discriminability (*d*’) and speeded reaction times. In contrast to aripiprazole’s neural effects, the risperidone group demonstrated a trend for reduced DLPFC recruitment. Unexpectedly, the risperidone group demonstrated similar effects to aripiprazole on *d*’ and additionally had reduced errors of commission compared with placebo.

**Conclusion:**

Aripiprazole has unique DLPFC actions attributed to its prefrontal D_2_ agonist action. Risperidone’s serotinergic action that results in prefrontal dopamine release may have protected against any impairing effects of its prefrontal D_2_ blockade.

**Electronic supplementary material:**

The online version of this article (doi:10.1007/s00213-016-4234-9) contains supplementary material, which is available to authorized users.

## Introduction

### Aim and rationale

The aim of this study was to investigate the implications of the partial dopamine D_2_ receptor agonist properties of aripiprazole for the neural basis of working memory in healthy volunteers. Impaired working memory is a core feature of schizophrenia which is not ameliorated by conventional antipsychotic drugs (Forbes et al. 2009). The impairment has been attributed to an impaired dopamine innervation of the dorsolateral prefrontal cortex (DLPFC) (Akil et al. 1999; Davis et al. 1991; Pycock et al. 1980; Slifstein et al. 2015; Winterer and Weinberger 2004), a key component of the neural circuitry of working memory. There has been much clinical interest in the possibility that the partial agonist actions of aripiprazole might restore D_2_ neurotransmission to a low optimal level in prefrontal cortex in schizophrenia (Bolonna and Kerwin 2005). This would preserve or enhance working memory while antipsychotic effects are achieved by preventing overstimulation of D_2_ receptors in striatum. We investigated the effects of aripiprazole on the performance of a working memory task and its neural correlates using functional magnetic resonance imaging (fMRI). We compared the effects of aripiprazole with those of risperidone since both drugs have high affinity for the D_2_ receptor combined with serotonergic effects although risperidone lacks the intrinsic D_2_ agonist activity of aripiprazole.

### Dopamine modulation of DLPFC and working memory

Working memory (WM) refers to the ability to hold and manage information ‘online’ over short periods of time, allowing its manipulation and usage in reasoning, comprehension and decision-making (Levy and Goldman-Rakic 2000). Numerous neuroimaging studies have shown that a network of cortical regions centred on the DLPFC is activated by performance of WM tasks such as the n-back task (see below). Impaired WM performance in patients with schizophrenia is associated with reduced DLPFC activation compared to controls (Carter et al. 1998; Perlstein et al. 2001; Weinberger et al. 1986). This is thought to reflect cortical inefficiency because greater DLPFC activation is elicited in patients when performance matches control levels (Callicott et al. 2000; Manoach et al. 1999, 2000).

Dopamine has an important role in WM performance in both non-human primates (Brozoski et al. 1979; Sawaguchi 2001; Sawaguchi et al. 1990) and in human volunteers (Egan et al. 2001). Dopamine modulates PFC activity by both D_1_ (Seamans and Yang 2004) and D_2_ receptor actions on pyramidal cells and interneurons (Wang et al. 2004), which influence different functional aspects of WM in experimental animals (Durstewitz and Seamans 2008; Seamans and Yang 2004). Studies in humans suggest a role for D_2_ receptors in working memory (Kimberg et al. 1997; Luciana et al. 1992; Luciana and Collins 1997; Mehta et al. 1999, 2001) although it is unknown whether these effects are driven by striatal or cortical D_2_ receptor actions given the systemic drug administration of these studies.

The foregoing evidence suggests that dysregulation of the DLPFC may contribute to the executive functioning impairments seen in schizophrenia and that impaired dopamine function could be a key mechanism. The actions of antipsychotic drugs on dopamine function within the dorsolateral prefrontal cortex are therefore of considerable interest.

### Neurochemical profiles of aripiprazole and risperidone

Aripiprazole is an atypical antipsychotic with a very high affinity (0.34 nM) (Burris et al. 2002) for the D_2_ receptor. In contrast to all currently available antipsychotic drugs, aripiprazole has a partial agonist effect on the D_2_ receptor with a range of intrinsic efficacies dependent upon the G protein-coupled receptor system it is acting upon (Lawler et al. 1999; Shapiro et al. 2003). Because of its high D_2_ affinity, aripiprazole is thought to clamp synaptic dopamine function at a constant but sub-maximal level of activation regardless of local dopamine concentration. This results in functional antagonism in areas of high dopamine concentration such as within the striatum. However, in areas of low dopamine concentration such as prefrontal cortex, aripiprazole’s partial agonism would exceed the local action of dopamine (Bolonna and Kerwin 2005; Grunder et al. 2003) resulting in increased D_2_ function. In addition to direct effects at D_2_ receptors, aripiprazole has been shown to increase dopamine release into the mouse prefrontal cortex in in-vivo microdialysis studies (Li et al. 2004; Zocchi et al. 2005). This effect may be due to the 5-HT_1A_ receptor partial agonism (*Ki* = 3.4 nM) and the 5-HT_2A_ antagonism (*Ki* = 4.2 nM) that aripiprazole also possesses (Stark et al. 2007) as both of these actions have been shown to increase prefrontal dopamine release (Li et al. 2004). Therefore, aripiprazole may also indirectly increase D1 neurotransmission during working memory by increasing prefrontal dopamine levels.

Risperidone has a high affinity for D_2_ receptors but acts as a pure antagonist. Furthermore, risperidone, like aripiprazole, increases dopamine release in the prefrontal cortex (Kuroki et al. 1999) and is a high-affinity 5-HT_2A_ receptor antagonist (*Ki* = 0.15–0.4 nM) (Leysen et al. 1994; Richelson and Souder 2000). By comparing the two drugs, we aimed to isolate aripiprazole’s D_2_ agonist effects from other actions that it shares with risperidone. This allowed us to separate out the functional importance of cortical versus striatal D_2_ receptors in working memory as both drugs reduce striatal D_2_ neurotransmission but only aripiprazole is hypothesised to have partial agonist effects at prefrontal D_2_ receptors. We hypothesised that aripiprazole would enhance or change prefrontal cortex WM activations in relation to improved performance compared to placebo and risperidone.

## Methods

### Participants

This study was approved by the NHS Research Ethics Committee and the Committee on the Ethics of Research on Human Beings of the University of Manchester. The study was therefore performed in accordance with the ethical standards laid down in the 1964 Declaration of Helsinki. Male participants were recruited via e-mail and were paid for their participation. Participants had to meet the following criteria: healthy according to physical examination including blood pressure, kidney and liver function tests and blood glucose levels, general health questionnaire and electrocardiography. Participants had no current diagnosis, or history of, psychiatric illness or substance use disorder. Participants were not taking any medication, were non-smokers and were right handed. Participants consumed under 20 units of alcohol a week and were asked to remain abstinent from alcohol and caffeine for 24 h prior to the study; compliance was self-reported.

Screening procedures included the Quick Test (Ammon and Ammons 1962) to provide an estimate of IQ, Mini International Neuropsychiatric Interview (MINI) (Sheehan et al. 1998), and the Edinburgh Handedness Inventory (EHI) (Oldfield 1971). The mean age (SD) was 23 (4.96), mean IQ (SD) was 94 (7.96), and the mean weight (SD) was 68.2 kg (11.6). ANOVAs revealed that there were no significant differences in age (*p* = 0.32), IQ (*p* = 0.97) or weight (*p* = 0.39) of the participants assigned to the placebo, aripiprazole or risperidone drug group. Urine drug tests, electrocardiograms (ECG) and blood tests were carried out at screening and any participants with a positive result and/or abnormal ECG reading or blood results were excluded from the study. Written informed consent was obtained from the participants before enrolment to the study.

## Experimental design and procedures

The study was performed following a randomised, double-blind, placebo-controlled, parallel group procedure. This parallel group design was chosen over a within-subject design to avoid practice effects on n-back performance and habituation effects to both the scanner environment and n-back task. Interactions between these effects and drug effects would make results difficult to interpret. Thirty-seven participants took part in the study in total: 13 participants received an acute oral dose of 5 mg aripiprazole, 12 received 1 mg risperidone, and 12 received placebo. Scanner technical problems resulted in loss of data from three participants (1 placebo, 1 aripiprazole, 1 risperidone). Therefore, three participants were excluded prior to any group analysis. The doses were subtherapeutic, chosen to minimise side effects in healthy volunteers. Recent studies have examined aripiprazole receptor occupancy in healthy volunteers after acute doses of aripiprazole; one study demonstrated that 5 mg of aripiprazole resulted in a mean striatal D_2_ occupancy of 55 % (Kim et al. 2012). Another study reported 74 % striatal and 51 % frontal D_2_ occupancy after 6 mg of aripiprazole (Takahata et al. 2012). A single 1 mg dose of risperidone results in striatal D_2_ occupancies of 50 % in healthy volunteers (Nyberg et al. 1993). Whilst this study did not measure extrastriatal D_2_ occupancy, PET studies assessing frontal occupancy after a single dose of 2 mg risperidone, and continuous dosing of 1 mg risperidone, suggest that frontal D_2_ occupancies of risperidone, whilst being slightly lower, do not differ significantly from striatal D_2_ occupancies (Agid et al. 2007; Ito et al. 2009). Taken together, it may be assumed that similar frontal D_2_ occupancies of slightly below 50 % were achieved with single doses of 5 mg aripiprazole and 1 mg risperidone.

Participants were scanned 3.5 h after taking the test drug as this represents the *tmax* for aripiprazole (Kubo et al. 2007). The *tmax* for risperidone and its equally potent metabolite is 1.5 and 3 h, respectively (Ereshefsky 1996; Huang et al. 1993). Participants were assessed for medication side effects by the on-site study clinician. Upon leaving the scanner, blood pressure was taken both standing and sitting and participants were assessed once more before being allowed to leave.

### N-back task

The task was programmed using E-Prime v1.1.4.4. SP3 (Psychology Software Tools 2003). Stimuli were presented in a block design, with three different conditions presented in 26-s blocks. For the 0-back condition, participants viewed a series of 13 letters which were presented with an interstimulus interval of 2 s (letter presented for 1500 ms followed by a blank screen for 500 ms). Participants were asked to respond with a button press on a button box whenever they saw an ‘X’ appear on the screen, this condition does not require any working memory processing and therefore represents the control condition for the task. For the 1-back condition, participants were asked to respond when they saw a letter which was the same as the last one presented. For the 2-back condition, participants were asked to respond when they saw a letter on screen that was the same as the one presented before the last letter. To avoid potential ceiling effects, participants were not permitted to practise the task although they received detailed task instructions and it was ensured they understood each task condition before entering the scanner. Thirteen letters were presented in each test block and each block contained three target stimuli. The task comprised three 2-back blocks, three 1-back blocks and six 0-back blocks presented in a pseudo-randomised order with a block of the 0-back condition presented between each 1-back and 2-back block. Whilst in the scanner, participants were instructed on the type of block they were about to perform by verbal instructions presented on the screen for 9 s.

Missed targets were recorded as omission errors (OEs), whereas participants’ responses to non-targets were recorded as commission errors (CEs). There were a total of 9 target letters for the 1-back and 2-back condition and 18 target letters for the 0-back condition. The task lasted 7 min.

### Statistical analysis of behavioural data

Participants made few errors of commission or omission. Chi-square tests (likelihood ratio; Lχ^2^) were used to assess whether there was an association between the drug taken and the frequency of OEs and CEs made for the 0- (baseline), 1- and 2-back conditions. Furthermore, discriminability or *d* prime (*d*’) was assessed. Hit rate was defined as the proportion of the targets responded to and the false alarm (FA) rate was the proportion of non-targets responded to. The hit rate and false alarm rate were transformed to Z-values using the NORMSINV function in excel (Haatveit et al. 2010). *d*’ is calculated with the following formula: *Z*_hit_ − *Z*_FA._ Perfect hit rate or FA rates of 1 or 0 respectively were converted using the following formulas before the Z-transform was carried out; proportions of 1 were converted to 1 − 1/(2*N*) and proportions of 0 were converted to 1/(2*N*) (Haatveit et al. 2010). A mixed ANCOVA using SPSS was carried out (n-back level and drug group as the within- and between-subject factor respectively) with the *d*’ score for the 0-back condition (which does not measure working memory but indexes attentional processing) added as a mean centred covariate.

Statistical analyses were carried out using SPSS v22. A mixed ANCOVA (n-back level and drug group as the within and between group factor respectively) was used to investigate drug effects on reaction times for correct responses for the 1-back and 2-back levels. To control for individual differences in motor responses, the reaction times for correct responses on the 0-back condition were entered as a mean centred covariate in the analysis. For *d*’ and reaction time analyses, main effects of group were investigated with Sidak post hoc tests, and drug group by n-back level interactions were investigated with both paired and two-sample *t* tests. A one-way ANOVA was carried out on the 0-back condition to test for any effects of the drugs on attentional processing.

Due to technical problems with recording responses, response data was lost from 3 out of the 12 participants in the aripiprazole group and a further 1 from the 11 participants in the placebo group; therefore, behavioural data from 9 participants from the aripiprazole group, 10 from the placebo group and 11 from the risperidone group were entered into the behavioural analysis.

### Image acquisition of functional data

Whole brain T2*-weighted images were acquired on a 3 T Philips *Achieva* scanner with single shot, gradient echo-planar imaging with the following parameters: FOV = 230 mm, acquisition matrix = 128 × 128, TR/TE 2000/35 ms, voxel size = 1.8 × 1.8 × 3.0 mm, 0.5-mm slice gap, and 34 slices. T1-weighted structural images were acquired for each participant in order to exclude patients with any anatomical abnormalities and for use during the preprocessing of functional images.

### Preprocessing and analysis of fMRI data

Final group size for fMRI data analysis was 12 for aripiprazole, 11 for risperidone and 11 for placebo. The functional images were converted from the PARREC format to the analyse format using MRIcro (version 1.40). Functional data were analysed using Statistical Parametric Mapping (SPM12; The Wellcome Department of Cognitive Neurology, London, England) implemented in Matlab (R2013a).

Images were realigned to correct for motion artefacts, and the T1 structural image was coregistered with the mean image of the realigned functional images. The T1 structural image was segmented and normalised using tissue probability templates supplied by SPM. Normalisation parameters were then applied to the functional images. Images were smoothed using a Gaussian kernel of the following dimensions 5.4 mm × 5.4 mm × 10.5 (three times the voxel size).

### First-level analysis

Data were fitted via general linear modelling in SPM (0-back, 1-back and 2-back conditions). The input model was convolved with the haemodynamic response function (HRF) in order to provide a better fit of the data (Smith 2001). The low-frequency drifts in the data were modelled out using a high pass filter set to 140 s, equivalent to two times the main repetition time of the task. The following contrasts were specified: 1-back minus 0-back, 2-back minus 0-back, 1-back&2-back minus 0-back and 2-back minus 1-back. Movement outliers (scan to scan displacement of >2 mm or volumes with global brain activation >3 standard deviations away from the mean) were detected using Artifact Detection Tools (ART) (http://www.nitrc.org/projects/artifact_detect/), and these outliers were added as nuisance regressors to the first-level model. All participants had less than 10 % of volumes detected as outliers.

### Second level analysis

As fMRI packages only produce one error term during statistical analysis, statistical tests containing both within- and between-subject factors within a single model are not validly performed (McLaren DG et al. 2011). For this reason, separate models were used for both within- and between-subject factors. To assess the effect of the n-back task and main effect of drug group, an ANOVA was specified with the average of the 1-back and 2-back condition (compared to 0-back) as a factor. To assess main effect of n-back level and n-back level by drug group interactions, an ANOVA was specified containing the 2-back minus 1-back contrast as a factor. Cluster thresholding was used with a cluster forming thresholded of *p* < 0.005. For the main effects of task and n-back level in all 34 participants, a whole-brain family-wise error cluster threshold of *p* < 0.05 was applied (*p*FWEc < 0.05). Assessments of drug group effects and n-back level by drug group interactions were confined to a DLPFC region of interest and thresholded at *p*FWEc < 0.05 small volume corrected (SVC). The DLPFC region of interest (ROI) comprised of a sphere of 10-mm radius centred around the Talairach coordinates (42, 32, 30) representing the peak DLPFC activation generated from a meta-analysis of verbal, identity-monitoring n-back task data in healthy controls (Owen et al. 2005). Furthermore, ROI analysis was carried out with and without a mask of the positive main effect of the n-back task (*p*FWEc < 0.05) to determine whether the drug effects occurred within regions activated by the task. The average of the beta-estimates were extracted from clusters significant for drug group effects (main effects or interactions) and plotted. Effects significant in the ANOVA were investigated with post-hoc two-sample *t* test comparisons.

To determine if any of the drug effects on brain activation influenced the behavioural measures, correlational analyses were carried out between BOLD signal change within the 1-back and 2-back conditions compared to 0-back and the behavioural measures (CE, OE, *d*’ and reaction time for correct responses), using any clusters showing significant drug effects as regions of interest. This was carried out within each drug group individually and with all participants from all three drug groups together.

## Results

### Tolerability

The medications were well tolerated. Mild nausea was reported by one participant taking aripiprazole although this was temporary and the participant was able to carry out the scan. There were no other adverse reactions to the medications, and no participants were excluded on the basis of lack of tolerability.

## Behavioural

### Omission and commission errors

Most participants made no CEs or OEs in the 0-back and 1-back condition. This pattern was not modified by drug treatment (Lχ^2^ not shown). More errors were made in the 2-back condition but OEs remained unaffected by drug treatment (Lχ^2^(6) = 9.92, *p* = 0.16; see supplementary materials for figure). In contrast, CEs at 2-back were affected by drug treatment (Lχ^2^(4) = 13.723, *p* = 0.013); only 3 of 11 participants treated with risperidone made CEs compared with 9/10 placebo and 7/9 aripiprazole treated participants.

### Discriminability *d*’

**Fig. 1:**
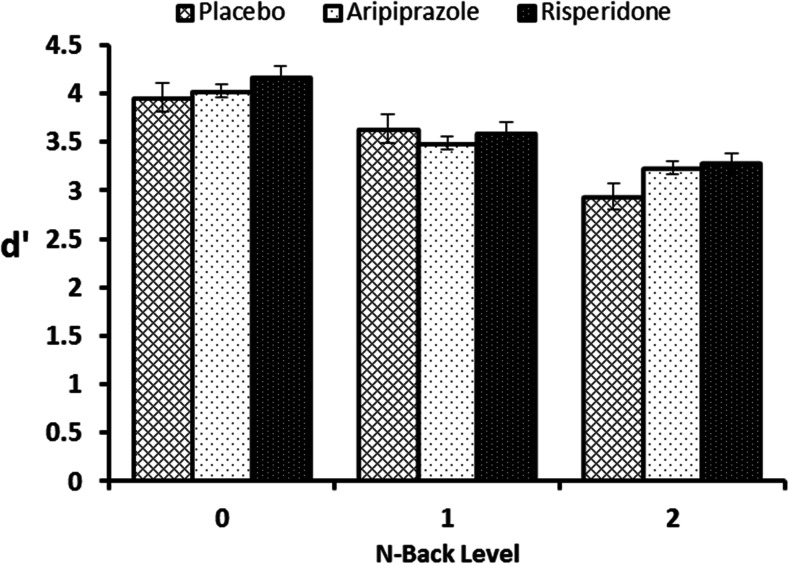
N-back d' scores. Scores shown for the 0-back (baseline condition), 1-back and 2-back condition for the placebo, aripiprazole and risperidone groups. Error bars indicate standard error of the mean

There were no significant effects of drug group on the 0-back condition, F(2,27) = 0.60, *p* = 0.55. The mixed ANCOVA revealed a significant effect of n-back, F(1,26) = 31.95, *p* < 0.001, and a significant drug by n-back level interaction, F(2,26) = 3.45, *p* = 0.047. From Fig. 1, it can be seen that the main effect is driven by a general lowering of *d*’scores in the 2-back condition compared to the 1-back condition, reflecting the greater task difficulty. However. post hoc paired *t* tests revealed the interaction to be caused by *d*’ scores being significantly different for the 2-back compared to 1-back for placebo (*p* < 0.001) and risperidone (*p* = 0.027), but not for the aripiprazole condition (*p* = 0.223). Furthermore, drug group *t* test comparisons at each of the two n-back levels (with 0-back covariate) revealed a trend for a significant difference between placebo and aripiprazole (*p* = 0.057) and risperidone and placebo (*p* = 0.054) for the 2-back condition but not for the 1-back condition (*p* > 0.1).

### Reaction times

**Fig. 2:**
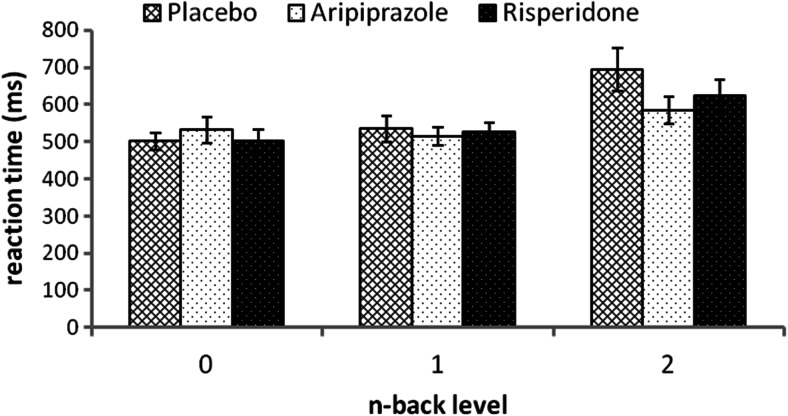
Reaction time for correct responses. Reactions times shown for 0-back (baseline condition), 1-back and 2-back condition of the n-back task for the placebo, aripiprazole and risperidone groups. Error bars indicate standard error of the mean

There was no significant effect of drug group on reaction times for correct responses for the 0-back condition, F(2,27) = 0.31, *p* = 0.74, indicating that there were no drug effects on motor speed or attention. The mixed ANCOVA revealed an overall significant effect of the 0-back reaction time covariate, F(1,26) = 25.407, *p* < 0.001, but no covariate by n-back level interaction, F(1,26) = 0.063, *p* = 0.804, indicating that reaction times for the 0-back condition were significantly related to reaction times for both the 1-back and the 2-back condition. The mixed ANCOVA revealed a significant effect of n-back level F(1,26) = 25.4, *p* < 0.001, but no n-back level by drug interaction, F(2,26) = 1.511, *p* = 0.24, indicating that reaction times slowed with increasing task difficulty although this occurred to the same degree across all treatment groups. Reaction times were faster after both drugs (notably aripiprazole) (Fig. 2), but this fell short of full statistical significance (F(2,26) = 3.006, *p* = 0.07). Sidak post hoc tests revealed a trend for faster reaction time with aripiprazole (*p* = 0.06) compared with placebo. No trends were found for the risperidone placebo comparison (*p* = 0.59) nor were there any trends for significant differences in the aripiprazole versus risperidone comparison (*p* = 0.44).

## fMRI

### Positive effect of n-back task and n-back level (cluster forming threshold of *p* < 0.005, *p*FWEc < 0.05)

The n-back task (1&2back across all groups) produced significant activations in the expected cortical areas for working memory tasks as identified in the normative meta-analysis of n-back task (Owen et al. 2005). These included the bilateral DLPFC, the bilateral ventrolateral prefrontal cortex (VLPFC), the bilateral premotor cortex, the bilateral rostral PFC, the bilateral cingulate gyri and the bilateral inferior and superior parietal cortices (Fig. S1 included in supplementary materials). Furthermore, all regions were recruited to a greater degree in the 2-back condition compared to the 1-back condition, reflecting the greater task difficulty.

### Main effects of drug and drug by n-back level interactions (cluster forming threshold of *p* < 0.005, *p*FWEc < 0.05, SVC)

**Fig. 3:**
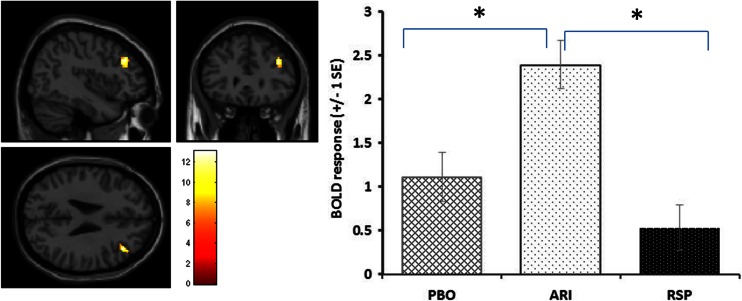
Brain images show the sagittal, coronal and axial views of the significant DLPFC cluster demonstrating a main effect of drug group (F-contrast image thresholded at *p* < 0.005). Histograms demonstrate the mean BOLD response within this cluster for the different drug groups. * = comparisons significant at *p*FWEc < 0.05 SVC for DLPFC in post hoc tests

Main effect of drug group was found within the DLPFC with an extent of 64 voxels at Talairach coordinates (42, 32, 28), *p*FWEc = 0.008 SVC for the DLPFC ROI. Masking with the positive effect of task revealed this drug effect to occur entirely within regions activated by the task. Post hoc two-sample *t* tests with the DLPFC ROI revealed that this effect was due to aripiprazole increasing activation within the DLPFC compared to both the risperidone group, *p*FWEc = 0.004 SVC, and the placebo group, *p*FWEc *=* 0.027. Compared to the placebo, there was a trend for a significant decrease with risperidone although this fell short of statistical significance, *p*FWEc = 0.07 SVC (also see Fig. 3)

No n-back level by drug interactions were found indicating that the drugs had similar effects during the 1-back and the 2-back conditions.

### Correlational analyses

None of the areas produced by the main effect of drug were found to be positively or negatively correlated with any of the behavioural measures for the 1-back minus 0-back or the 2-back minus the 0-back conditions.

## Discussion

The aim of this study was to determine whether partial agonist effects of aripiprazole on dopamine D_2_ receptors in the prefrontal cortex would improve performance of a working memory task in healthy volunteers in comparison with neutral or adverse effects of the full D_2_ receptor antagonist risperidone. As hypothesised, aripiprazole enhanced activation in the DLPFC and demonstrated a trend for improved reaction times and discriminability (*d*’) compared to placebo. Risperidone reduced activation within the right DLPFC compared to aripiprazole with a trend for reduced activation compared to placebo. However, against our prediction, the risperidone group had reduced errors of commission and had similar effects to aripiprazole on *d*’. Aripiprazole and risperidone have direct actions at D_2_ receptors and serotonin receptors, but they are also expected to have indirect actions at D_1_ receptors due to their serotonin receptor–mediated increases in prefrontal dopamine release (Kuroki et al. 1999; Li et al. 2004). Therefore, the fMRI and behavioural effects of the study drugs are discussed in terms of actions at D_2_, D_1_, and 5-HT receptors.

### Dopamine modulation of the prefrontal cortex during working memory

Dopamine is released in the prefrontal cortex during the performance of working memory tasks (Watanabe et al. 1997) where it acts to modulate the activity of PFC neurons (Durstewitz et al. 2000; Williams and Goldman-Rakic 1995). Studies have demonstrated that direct application of D_1_ (Sawaguchi 2001; Sawaguchi and Goldman-Rakic 1991, 1994) and D_2_ drugs (Wang et al. 2004) modulate prefrontal neuronal activity during working memory performance in non-human primates. Studies in humans demonstrate that D2 receptors (Kimberg et al. 1997; Luciana and Collins 1997; Luciana et al. 1992; Mehta et al. 1999, 2001; Tarantino et al. 2011) modulate working memory performance and prefrontal activation (although the striatal vs prefrontal contribution to these is unknown). Electrophysiological studies demonstrate that D_1_ and D_2_ receptors influence prefrontal cortex pyramidal neurons in different ways (Seamans and Yang 2004) which may explain the behavioural and fMRI effects of aripiprazole and risperidone. Both antipsychotic drugs cause the release of dopamine into the prefrontal cortex (Li et al. 2004) therefore, both drugs are expected to have indirect D_1_ receptor effects. Given the D_2_ partial agonist effects of aripiprazole versus the D_2_ antagonism of risperidone, we expect aripiprazole to have both D_2_- and D_1_-mediated agonist PFC actions whereas we expect risperidone’s dopaminergic actions to be mediated by D1 receptor agonism.

### D_2_-mediated effects within the prefrontal cortex during performance of the n-back task

The n-back task requires participants to attend to newly presented letters whilst maintaining a representation of the letter that was presented ‘*n*’ letters back. The letter presented *n* trials back quickly becomes irrelevant as the next letter is presented, and the representation of this letter must be quickly replaced with newly presented letters. In contrast to delayed response tasks whereby a single stimulus needs to be maintained over a delay, items maintained in the n-back task are constantly changing, and for successful performance, new information needs to be readily incorporated into the PFC. D_2_ agonists have been shown to increase working memory capacity (the number of new items than can be encoded) whereas D_1_ effects are crucial for tasks involving the maintenance of a stimulus over a delay (Tarantino et al. 2011). D_2_ and D_1_ receptors have opposing effects on excitatory and inhibitory neurotransmission within the PFC. Studies have shown that D_2_ receptors decrease, and D_1_ receptors increase, NMDA (Zheng et al. 1999) and GABA currents (Kroener and Lavin 2010; Seamans et al. 2001). According to computational models, D_2_ receptors decrease the competition between groups of neurons within the PFC. These actions facilitate movement along representations, aiding the fast switching of attention and incorporation of new information into PFC networks (Durstewitz and Seamans 2008). Thus, aripiprazole’s partial D_2_ agonism may have contributed to the trends for speeded reaction time and improved *d*’. In a study examining executive functioning in healthy volunteers, the D_2_ agonist bromocriptine speeded reaction time in a Stroop task (i.e. decreased interference) (Roesch-Ely et al. 2005). The authors explain this effect in terms of D_2_-receptor-mediated facilitation of the switching from reading the colour of words to naming them. Too much D_2_ to D_1_ receptor stimulation can be detrimental to cognitive performance by increasing distractibility (Durstewitz and Seamans 2008) as demonstrated by the cognitive impairing effect of the D_2_ agonist bromocriptine in individuals with a high baseline working capacity who are hypothesised to have optimum dopamine levels at baseline but are ‘overdosed’ after bromocriptine administration (Gibbs and D’Esposito 2005; Kimberg et al. 1997). Therefore, a combination of aripiprazole’s partial, rather than full, D_2_ agonism, along with its secondary D_1_ receptor effects, as aripiprazole has been shown to reverse cognitive deficits in rodents via D_1_ receptor agonism (Nagai et al. 2009), may contribute to its favourable effects on reaction time. Notably, however, a very recent study assessing the acute effects of 10 mg aripiprazole on n-back performance in healthy volunteers found contradictory results to the present study in that they reported *increased* reaction times with aripiprazole and no change in neural activation compared to placebo (Goozee et al. 2015). We believe that dose differences underlie these contradictory findings. Kim et al. compared resting brain metabolism, receptor occupancy and n-back performance in healthy volunteers taking various doses of aripiprazole (Kim et al. 2013). They demonstrated that both 10- and 30-mg doses significantly decreased resting brain metabolism whereas 2 and 5 mg of aripiprazole did not. Furthermore, frontal brain metabolism was found to be inversely correlated with striatal D2 receptor occupancy and n-back reaction time. These and our findings suggest a dose dependency of the cognitive effects of aripiprazole in healthy volunteers. Doses above 10 mg may have detrimental effects related to excessive striatal D2 blockade and reduced frontal metabolism. Doses of 5 mg may have beneficial effects which we hypothesise to be due to a combination of D2 and D1 prefrontal agonism and insufficient striatal D2 blockade to cause impairment.

Studies in schizophrenia patients support a cognitive enhancing role of higher doses (10 mg and above) of aripiprazole during working memory and verbal cognitive functioning (Bervoets et al. 2012; Kern et al. 2006; Maat et al. 2014; Schlagenhauf et al. 2010), although not all studies support a working memory enhancing role of aripiprazole (Riedel et al. 2010). Given that schizophrenia is associated with excessive striatal dopamine transmission (Howes et al. 2012), higher doses of aripiprazole may be tolerated before striatal D2 neurotransmission is reduced to detrimental levels.

The increased DLPFC activation with aripiprazole may be due to D_2_ receptor actions to reduce GABA_A_ inhibition of pyramidal neurons (Seamans et al. 2001). Whilst D_1_ receptor actions can spatially tune activation within the prefrontal cortex by increasing the inhibition of pyramidal neurons (Williams and Goldman-Rakic 1995), D_2_ receptor activation will reduce this inhibition, possibly allowing a greater extent of DLPFC to be recruited by the task. However, this explanation may be somewhat speculative when considering the BOLD signal reflects neuronal mass action (Logothetis 2008), whereas the above studies of Williams and Goldman Rakic and those of Seamans and colleagues report effects in individual neurons. Furthermore, D_2_ enhancements of prefrontal GABA neurotransmission have also been reported (Tseng and O’Donnell 2007). The increased BOLD signal in the DLPFC with aripiprazole was associated with a trend for faster reaction times and an improvement in *d*’, arguing against the suggestion that aripiprazole reduces neuronal efficiency, but rather that this enhancement had a functionally beneficial effect. This is in agreement with a recent finding in schizophrenia patients demonstrating greater PFC activation and improved performance on the n-back task after switching from typical antipsychotic medications to aripiprazole (Schlagenhauf et al. 2010).

### D_1_ receptor effects during the n-back task

Against our hypotheses, risperidone had similar effects to aripiprazole on ‘d, although unlike the aripiprazole group, this was driven by a reduced tendency to make commission errors, with minimal effects on reaction time. The lack of cognitive impairment after risperidone in this study is likely due to the low dose used (with predicted D_2_ occupancy of 50 %). We and others have reported that single doses of risperidone at 2-mg impair reaction time and performance in healthy volunteers (Koychev et al. 2012). However, studies in clinical populations have also failed to demonstrate a superiority of aripiprazole over risperidone for cognitive functioning (Khanna et al. 2013, 2014). This suggests that there are protective factors associated with risperidone’s mode of action that compensate for its high-affinity D2 blockade. Although both drugs release PFC dopamine (due to their serotinergic actions), risperidone may do this to a greater degree given its higher 5-HT_2A_ receptor affinity. Reduced CEs after risperidone may reflect a combination of increased dopamine release acting on D_1_ receptors with insufficient D_2_ blockade to interfere with performance. Computational accounts suggest that D_1_ receptors increase signal and reduce noise through simultaneously enhancing glutamatergic maintenance of representations while enhancing GABA surround inhibition (Durstewitz and Seamans 2002; Williams and Castner 2006). D_1_-mediated improved signal to noise could thus account for the improved performance and the trend for reduced cortical BOLD response after risperidone—the latter reflecting more efficient cortical processing. Indeed, dopamine-releasing drugs reduce PFC metabolic demand and BOLD signal in healthy volunteers during the performance of cognitive tasks, both in the context of improved performance and in the absence of a performance effect (Mattay et al. 2003; Mehta et al. 2000; Tipper et al. 2005; Volkow et al. 2008).

### Direct actions at serotonin receptors

A reason for the lack of performance differences between aripiprazole and risperidone (and the lack of differences in cognitive effects found clinically) could be the serotonergic actions of risperidone. The effects of 5-HT_2A_ antagonists on neuronal activity appear to be similar to that of D_2_ agonism of reducing pyramidal and interneuron activity to decrease signal to noise during delayed response tasks (Williams et al. 2002). Direct application of a 5-HT_2A_ receptor antagonist into the PFC reduces impulsive responding in rats (Winstanley et al. 2003); therefore, risperidone may be reducing commission errors via a reduction in impulsivity. A recent computational model suggests that 5-HT_1A_ receptor activation has functional actions within the PFC during working memory, again similar to effects expected from D_2_ activation (Cano-Colino et al. 2013). Therefore, some of aripiprazole’s fMRI and behavioural effects may be the result of a combination of D_2_ agonism, 5-HT_2A_ antagonism and 5-HT_1A_ agonism.

### Correlations between behavioural data and BOLD signals

No positive or negative correlations were found between task performance and activity within the DLPFC. A likely reason is that each drug affects several neural and cognitive processes that influence performance which lack a final common, performance-related effect on DLPFC neuronal activity and resultant BOLD signal. Risperidone appeared to increase neural efficiency in the DLPFC since reduced activation was associated with enhanced performance, whereas aripiprazole appeared to increase the recruitment of DLPFC neurons to aid performance. The n-back task requires the execution of numerous cognitive operations, and therefore different strategies are likely to be used by different individuals to complete the task, which could explain why correlations were not found (Rypma et al. 2006). Furthermore, ceiling effects may have resulted in insufficient performance variations to allow for correlations to be found.

### Limitations

The main limitation of this study is the low sample size which resulted in low statistical power. This may have contributed to the majority of the performance findings being of only trend-level significance. Ceiling effects may also have constrained detection of performance-enhancing effects of aripiprazole. Indeed, other studies have reported drug-induced behavioural differences in healthy volunteers only in the 3-back version of the task (Mattay et al. 2000, 2003). Similarly, near-perfect accuracy in the controls reduced the possibility of detecting improved accuracy after drug treatment. Nevertheless, the data suggest the drugs may improve performance in different ways—aripiprazole increased arousal (faster reaction times), whereas risperidone reduced commission errors but had a reduced benefit on arousal.

Another limitation associated with the low statistical power of the study may be that effects meeting statistical significance (such as the fMRI effects in this study) may represent an overestimation of the ‘true’ effect (Button et al. 2013).

The use of healthy volunteers rather than schizophrenia patients could be seen as a limitation. However, risperidone has been shown to both improve (Green et al. 1997) and impair (Reilly et al. 2006, 2007) performance of a working memory task in schizophrenia patients. The contrasting results are likely to have been due to differences, possibly unknown, in illness and medication status of the patients. Such confounds are by-passed in experiments in healthy volunteers.

Another potential limitation is that low doses of the study drugs were used to minimise side effects, and therefore study findings may not be applicable to the recommended doses for the treatment of schizophrenia. As discussed, there appears to be markedly different effects of aripiprazole and risperidone in healthy volunteers, depending upon the dose used. Therefore, it is quite possible that any beneficial effects of these drugs at the doses used in this study may disappear at recommended therapeutic doses, possibly explaining the limited effects of these drugs on working memory in a study of schizophrenia patients (Riedel et al. 2010). However, 5 mg of aripiprazole was shown to significantly reduce negative symptoms in a double-blind placebo controlled study in acutely relapsing schizophrenia patients (Cutler et al. 2006). Furthermore, low doses of aripiprazole and risperidone are efficacious as adjunct treatments for major depressive disorder, an indication for which aripiprazole is FDA-approved (Berman et al. 2007; Mahmoud et al. 2007; Marcus et al. 2008; Terao 2008). Therefore, the doses used in the study have clinically significant effects that may be mediated by the prefrontal actions demonstrated in this study. Although the degree to which the single dose used in the current study can be applicable to effects observed after repeated dosing is unknown, we feel that the finding that acute low doses of antipsychotic drugs do not impair but may potentially enhance cognitive function is of clinical interest.

## Conclusion

Aripiprazole increased DLPFC activation during the n-back task, and this was associated with a trend for faster performance and improved discriminability compared to placebo. Risperidone also tended to improve discriminability and reduced incorrect responding which was associated with a trend for reduced recruitment of the DLPFC compared to placebo. The results are compatible with computational models according to which (i) partial D_2_ agonist effects of aripiprazole would enhance working memory capacity and thus processing speed whereas (ii) D_2_ receptor blockade after risperidone together with increased dopamine release onto D_1_ receptors would enhance signal to noise and thus cortical efficiency and performance accuracy. Probably more complex interactions between dopamine and serotonin play a role in the contrasting actions of the drugs.

## Electronic supplementary material

Below is the link to the electronic supplementary material.

ESM 1 (RTF 13397 kb)
