# Supplementary material for: An investigation into aripiprazole’s partial D2 agonist effects within the dorsolateral prefrontal cortex during working memory in healthy volunteers
Source: Psychopharmacology (Berl). 2016 Feb 22;233:1415–26. doi: 10.1007/s00213-016-4234-9 (PMC4819596; doi:10.1007/s00213-016-4234-9)
Supplement: Supplementary file 1 — (RTF 13397 kb) [file 213_2016_4234_MOESM1_ESM.rtf]

Supplementary materials
An Investigation into Aripiprazole's Partial D2 Agonist Effects Within the Prefrontal Cortex During Working Memory in Healthy Volunteers
Anna Murphy, Serdar Dursun, Shane McKie, Rebecca Elliott, John Francis William Deakin
Journal: Psychopharmacology
Corresponding Author
Anna Murphy
Neuroscience and Psychiatry Unit
G.708 Stopford Building
University of Manchester, 
M13 9PT
anna.murphy@manchester.ac.uk
+44 (0)161 275 7764
Fax: +44 (0)161 275 7429
